# Pirated Inventions

**Published:** 1893-10-21

**Authors:** 


### EDITOR'S LETTER-BOX.

[Our correspondents are reminded that proxility is a great bar to publication, and that brevity of style and conciseness of statement greatly facilitate early insertion.]

### PIRATED INVENTIONS.

Sir,—May we ask your kind consideration of a matter which has for a very considerable time caused us great loss and annoyance, and not only us, but many other members of our trade? It is, namely, the indiscriminate and very unfair publication of so-called new inventions which have not an iota of claim to such appellation. It seems to us that so totally unfair a system can readily be changed by giving manufacturers an opportunity of offering their remarks to the Editor on any assumed new invention before the same is published. In case prior claim exists, such would naturally have to be accompansed by absolute proof. We are in a position to give numberless proofs of our assertion, but hardly think that there is any necessity for so doing.—We have the honour to be, Sir, your obedient servants,

John Weiss and Son (J. F. Fream).

287, Oxford Street, W.

*** We shall be glad to have our attention called to definite instances of the abuses complained of.—Ed. *T.H.*

